# Additive antidepressant‐like effects of fasting with β‐estradiol in mice

**DOI:** 10.1111/jcmm.14434

**Published:** 2019-06-18

**Authors:** Pu Wang, Bingjin Li, Jie Fan, Kun Zhang, Wei Yang, Bingzhong Ren, Ranji Cui

**Affiliations:** ^1^ School of Life Sciences Northeast Normal University Changchun P. R. China; ^2^ Jilin Provincial Key Laboratory on Molecular and Chemical Genetics The Second Hospital of Jilin University Changchun People's Republic of China

**Keywords:** antidepressant, fasting, RNA‐seq, β‐estradiol

## Abstract

Our recent study has shown that acute fasting produces antidepressant‐like effects in male mice. However, there is little evidence regarding the antidepressant‐like effects of acute fasting in female mice. Moreover, it is not yet clear whether estrogen produces additive effects with acute fasting. Therefore, this study aims to investigate the antidepressant‐like effects of acute fasting plus estrogen treatment. In this study, the acute fasting produced antidepressant‐like effects in female mice and the antidepressant‐like effects of 9 hours fasting with those of β‐estradiol (E2) were additive. Activity of the cyclic adenosine monophosphate (cAMP) response element‐binding protein (CREB)‐brain‐derived neurotrophic factor (BDNF) pathway in the prefrontal cortex (PFC) and hippocampus (HP) was increased, as well as neurogenesis in the subgranular zone of the hippocampus. Serum ghrelin and estrogen were also increased by fasting plus E2. Furthermore, RNA‐seq analysis indicated that fasting and E2 co‐regulate similar gene expression pathways, underlying similar neurological functions. Taken together, these data suggest that E2 produces additive antidepressant‐like effects with fasting by activating the CREB‐BDNF pathway in the PFC and HP. Genome‐wide transcriptome mapping suggests that fasting may be used as an adjunct to estrogen replacement therapy for the treatment of depression associated with reduced estrogen function.

## 1 INTRODUCTION

Calorie restriction has been reported to have anti‐ageing effects and to play an important role in several neurological diseases.^1^, ^2^, ^3^, ^4^ Recently, dietary restriction has received much attention in psychiatry due to its antidepressant and anxiolytic effects.^5^, ^6^ Our previous studies revealed that acute fasting for 9 hours, but not 3 hours or 18 hours, produces antidepressant‐like effects in the forced swimming test (FST). We also found that 9 hours of fasting combined with imipramine (30 mg/kg, ip) treatment produced additive antidepressant‐like effects in the FST and increased the p‐CREB/CREB ratio.^7^ However, the mechanisms of the antidepressant‐like effects of acute fasting remain unclear.

There are sex differences in depression.^8^, ^9^, ^10^ Women are more likely to develop depressive disorders than men,^10^ and the occurrence of depression in women is affected by hormone changes, for example reduced estrogen levels in menopause.^9^ Several studies have reported that estrogen replacement therapy exerts antidepressive actions in females with depression in clinical studies, as well as females in animal models.^8^, ^11^ Furthermore, estrogen can be combined with selective serotonin re‐uptake inhibitors (SSRIs), to produce additive antidepressant‐like effects.^12^, ^13^ Curiously, fasting produces estrogenic effects even in ovariectomized mice,^14^ and caloric restriction increases expression of estrogen receptors (ERs) but not the expression of the androgen receptors in mouse ovaries.^15^ Therefore, this study investigated the antidepressant‐like effects of acute fasting combined with estrogen treatment, and the potential mechanisms that might underlie these combined antidepressant effects.

## 2 MATERIALS AND METHODS

### 2.1 Animals

ICR (strain of albino mice originating in Switzerland, mice of this strain have been sent to various places from the Institute of Cancer Research in the USA, the strain was named ICR after the initial letters of the institute) female mice (8‐12 weeks) were obtained from Jilin University (Changchun, China). Mice were individually housed in plastic cages (25.5 × 15 × 14 cm), with food and water available ad libitum. The mice were maintained in standard laboratory conditions: 23 ± 1°C and 12 hours light/dark cycle (lights on/off at 7:00 am/ 7:00 pm). All behavioural studies were conducted during the light phase. All experiments were conducted in accordance with the Chinese Council on Animal Care Guidelines.

### 2.2 Drugs

E2 was purchased from Sigma Aldrich Co. (St. Louis, MO, USA) and dissolved in 5% arabic gum. The estrogen receptor antagonist tamoxifen (TMX) was purchased from Sigma Aldrich Co. (St. Louis, MO, USA), and dissolved in 10 µg/µL sesame oil. All drugs were administered intraperitoneally in a constant volume of 10 mL/kg body weight. To compare the effect of fasting and other drugs, mice were treated with either the respective vehicle or the respective drugs, E2 (15 μg/kg, ip) or TMX (15 mg/kg ip).

### 2.3 Open field test

For the open field test, mice were tested following our previous report 16 after 9 hours fasting. Mice were individually placed in a round acrylic apparatus (48.8 cm in diameter × 16 cm height wall) with a grey floor divided into 19 equal squares. The mice were placed in the centre of the apparatus for 6 minutes and recorded by an overhead digital camera. Horizontal locomotor activity (grid lines crossed with all four paws) and vertical locomotor activity (rearing) were determined by an observer that was blind to the treatment conditions. The apparatus was cleaned with 70% alcohol before each successive test. Results are expressed as mean ± SEM.

### 2.4 Forced swimming test

The forced swim test was performed after open field test in the same manner as our previous report.^16^ The mice were placed individually into a cylindrical container (height: 25 cm, diameter: 11 cm) filled with water (25°C ± 1°C) to the height of 20 cm. The test was recorded by video camera for 6 minutes. After swimming, mice were removed from the glass cylinder and dried with a towel and kept warm before returning them to their home cages. Behavioural observations were made of the immobility time during the last 4 minutes (240 seconds) of the 6 minutes testing period (mean ± SEM) by observers that were blind to the treatment conditions.

### 2.5 Protein analysis

#### 2.5.1 Sample collection

The animals were killed by decapitation immediately after behavioural testing. The PFC and HP were dissected and homogenized in lysis buffer (promega). The homogenates were centrifuged at 10,000 rpm, 4°C for 20 minutes. The supernatant was collected and stored at −80°C until use.

#### 2.5.2 SDS‐PAGE and Western blot analysis

The samples were separated on 10% SDS‐PAGE gels. The membranes were incubated with 5% non‐fat milk in TBST at room temperature and then incubated with primary antibodies overnight at 4°C with gentle shaking: (a) CREB (1:1000, rabbit monoclonal; Cell signalling, CST9191S); (b) Ser133‐phosphorylated CREB (p‐CREB) (1:1000, rabbit polyclonal; Cell signalling, CST9197S); (c) BDNF (1:1000, rabbit polyclonal; Santa Cruz Biotech, SC 546); (d) β‐actin (1:2000, Mouse monoclonal; TransGen Biotech; #HC201). The membranes were then washed with TBST and incubated with goat anti‐rabbit lg G‐HRP conjugated secondary antibody (Proteintech, SA00001‐2; CREB (1:2000), p‐CREB (1:1500), BDNF (1:6000)) at room temperature for 30 minutes. The membranes were visualized by the ECL reagents after exposing the membranes to X‐ray film in a dark room. Western blots were scanned and quantified by densitometry, using Image J software.

### 2.6 Serum corticosterone, ghrelin and estradiol analysis

Serum concentrations of CORT, ghrelin and E2 were quantified using a commercial enzyme‐linked immunosorbent assay (ELISA: Jiancheng Bioengineering, Nanjing, China) kit. Forty microlitres of samples were added to pre‐coated wells, followed by 10 μL of antibody and 50 μL streptavidin‐HRP, and incubated for 1 hour at 37°C. After the last wash, 50 μL of substrate A and 50 μL of substrate B were added to each well and mixed with gentle shaking for 10 minutes at 37°C without light. Fifty microlitres of stop solution was added to stop the reaction, and the optical density of the wells was measured at 450 nm. The concentrations were determined from the regression line for a standard curve.

### 2.7 Immunofluorescence histochemistry

Mice were administered 150 mg/kg (ip) 5‐Bromo‐2´‐deoxyuridine (BrdU, from SIGMA) on day 1 and 50 mg/kg on day 2. Mice were transcardially perfused with ice‐cold saline followed by 4% paraformaldehyde 2 hours after the last BrdU injection. Brains were dissected by vibratome (Leica, CM1860) coronally on a cryostat into 20‐μm‐thick sections containing the HP. Sections were incubated for 2 hours in 50% formamide/standard saline citrate (SSC) at 65°C; washed with SSC and incubated for 30 minutes in 2 N HCL/PBS, then for 25 minutes in 0.1 M boric acid. The sections were removed and placed in 3% normal goat serum in PBST (10 mM PBS with 0.3% Triton X‐100) for 1 hour, and then incubated in a rat anti‐BrdU monoclonal antibody (1:800; Abcam; ab6326) overnight at 4°C. The sections were then washed in PBST and incubated with an Alexa Fluor^®^ 488 goat anti–rat IgG antibody (1:500; molecular probes; A11006) for 2 hours at room temperature. The sections were then washed in PBS and a fluorescence microscope (Olympus, Tokyo) used to visualize the sections at a magnification of × 400.

### 2.8 RNA‐isolation, sequencing and bioinformatic analysis

PFC and HP tissues were rapidly dissected and frozen on dry ice. Total RNA was extracted from the samples using Trizol. Total RNA was equally mixed and submitted to Sangon Biotech (Shanghai) Co., Ltd. for 150 bp paired‐end reads sequencing on an Illumina HiSeq XTen platform (Illumina, San Diego, CA). Bioconductor software package for differential expression analysis of replicated count data was used to correct for multiple testing (false discovery rate (FDR) cutoff of <0.1) and for the identification of differentially expressed transcripts based on count per million (CPM) values. Genes with *P* < 0.05 were accepted to be differentially expressed. Furthermore, we performed a GO enrichment analysis usinghttp://www.geneontology.org/.^17^ For all analysis, the enrichment threshold was *P < *0.05.

### 2.9 Statistical analysis

All values are presented as the mean ± SEM Statistical analysis was performed using two‐way ANOVA with Factor 1 diet (fasting vs. non‐fasting) and Factor 2 treatment (veh vs E2) or one‐way ANOVA followed by Tukey's *post hoc* tests. Bioinformatics analysis was performed using Student's *t *test. *P* values less than 0.05 were considered significant.

## 3 RESULTS

### 3.1 Effects of acute fasting, E2 and TMX in the forced swimming test and open field test

**Figure 1:**
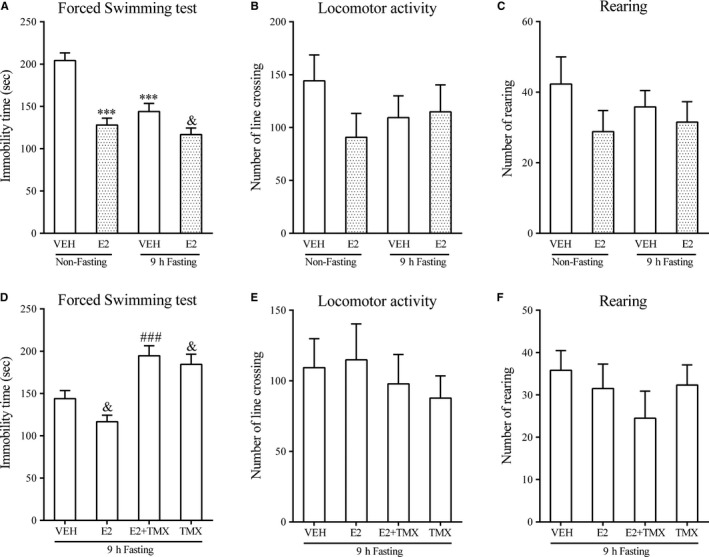
Effects of acute 9 h fasting, E2 and TMX on immobility time in the FST and locomotor activity in the OPT. FST: A and D; OFT: B, C, E and F. N = 6 per group. Data represented as mean ± SEM were analysed by two‐way (diet × treatment) ANOVA followed by *post hoc* comparisons for the data presented in A, B and C, or by one‐way ANOVA followed by *post hoc* analysis for the data presented in D, E and F. *** *P* < 0.001 compared with the non‐fasting + vehicle group; & *P* < 0.05 compared with the fasting + vehicle group; ### *P* < 0.001 compared with the E2 + fasting group. VEH: vehicle; E2: 17 β‐estradiol; TMX: tamoxifen

A two‐way ANOVA revealed a significant effect of estrogen treatment, fasting, and the interaction between estrogen treatment and fasting (*F*_diet_ *_(1,20)_* = 20.889, *P* < 0.001; *F*_treatment_ *_(1,20)_* = 43.770, *P* < 0.001; *F*_diet × treatment_ *_(1,20)_* = 9.744, *P* < 0.01，Figure 1A). *Post hoc* comparisons between groups showed that immobility time was significantly reduced by 9 hours of fasting compared with the non‐fasting/VEH group (*P* < 0.001). Administration of E2 (15 µg/kg) reduced the immobility time in both non‐fasting and fasting mice (*P* < 0.001, non‐fasting; *P* < 0.05, fasting), although to a lesser degree in fasting mice which accounted for the significant interaction. Figure 1D shows similar additive antidepressant‐like effects of estrogen in fasting mice that was reversed by administration of TMX (*F_(3,20)_* = 14.46, *P* < 0.001; *post hoc P < *0.001). TMX also reversed the effect of fasting alone on immobility time in the forced swim test (*P* < 0.05). In both cases, TMX treatment brought immobility scores back to the level of control mice (about 200 seconds; Figure 1A).

These effects seemed to be behaviourally selective. Figure 1B,C,E,F show that there were no changes in locomotor activity or rearing in the open field test in non‐fasting or fasting mice. Neither fasting nor E2 had any significant effect on the number of line crosses.

### 3.2 Effects of fasting, E2 and TMX on total CREB and p‐CREB protein levels in the PFC and HP

**Figure 2:**
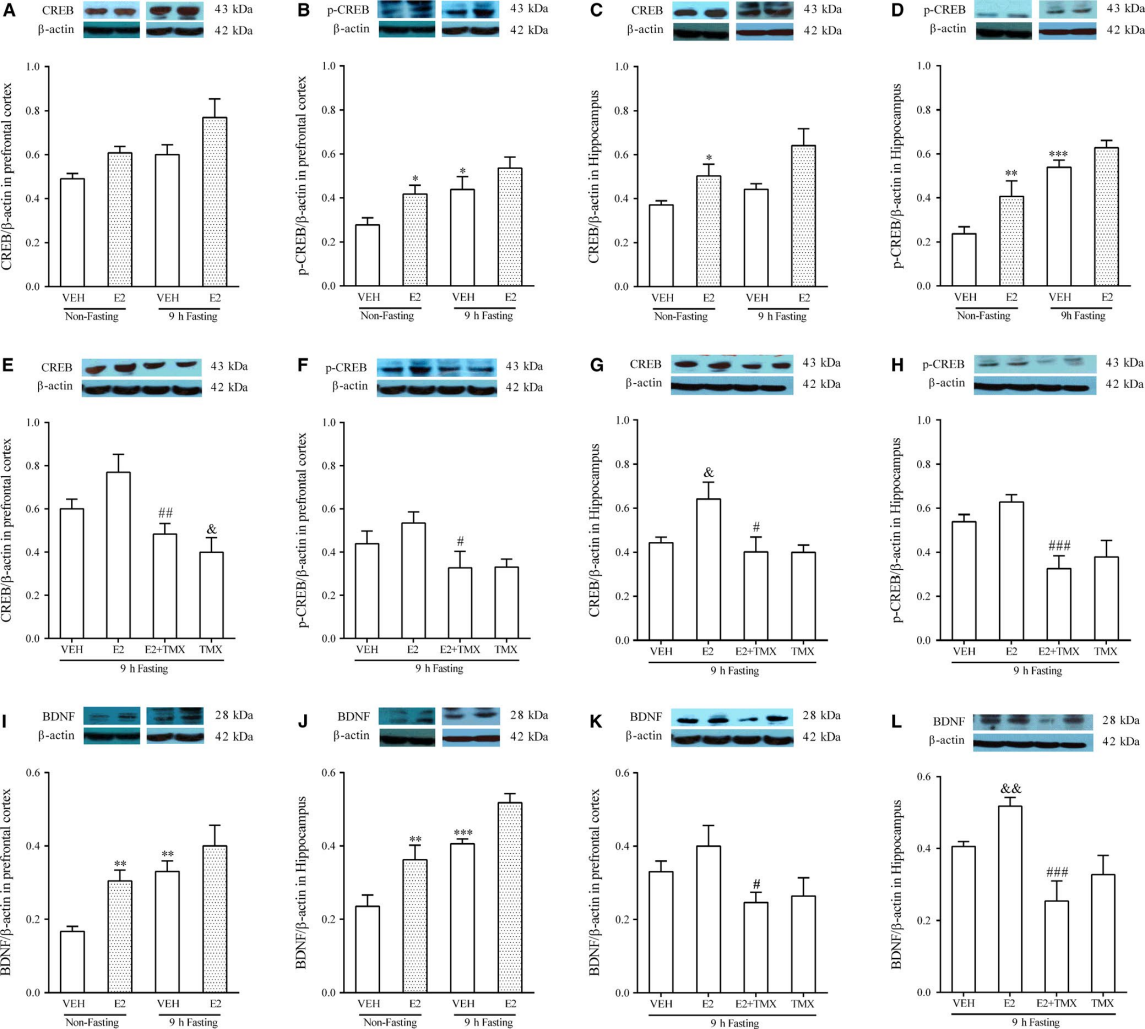
Effects of acute 9 h fasting, E2, and TMX on the CREB/BDNF signalling pathway in the PFC and HP. N = 6 per group. A, E: CREB/ β‐actin in PFC. B, F: p‐CREB/ β‐actin in PFC. C, G: CREB/ β‐actin in HP. D, H: p‐CREB/ β‐actin in HP. I, K: BDNF/ β‐actin in PFC. J, L: BDNF/ β‐actin in HP. Data represented as mean ± SEM were analysed by two‐way (diet × treatment) ANOVA followed by pairwise comparisons for the data presented in A‐D and I, J or one‐way ANOVA followed by *post hoc* analysis for the data presented in in E‐H and K, L. * *P* < 0.05; ** *P* < 0.01; *** *P* < 0.001 compared with the non‐fasting + vehicle group; & *P* < 0.05, && *P* < 0.01 compared with the fasting + vehicle group. # *P* < 0.05; ## *P* < 0.01; ### *P* < 0.001 compared with the E2 + fasting group. VEH: vehicle; E2: 17 β‐estradiol; TMX: tamoxifen.

Western blotting results for CREB and p‐CREB in PFC are shown in Figure 2A,B. Both E2 treatment and 9 hours fasting increased the levels of CREB and p‐CREB in the PFC (CREB: *F*_diet_ *_(1,20)_* = 8.276, *P* < 0.01; *F*_treatment_ *_(1,20)_* = 9.261, *P* < 0.01; *F*_diet × treatment_ *_(1,20)_* = 0.302, *P* = 0.588 in Figure 2A; p‐CREB:*F*_diet_ *_(1,20)_* = 10.608, *P* < 0.01; *F*_treatment_ *_(1,20)_* = 7.687, *P* < 0.05; *F*_diet × treatment_ *_(1,20)_* = 0.266, *P* = 0.612 in Figure 2B)_._ *Post hoc* comparisons between groups showed p‐CREB levels in PFC were significantly increased by either E2 or 9 hours fasting compared with the non‐fasting control group (*P* < 0.05 for E2; *P* < 0.05 for 9 hours fasting in Figure 2B). As shown in Figure 2E,F, increasing effects of the CREB and p‐CREB levels with E2 in fasting mice were reversed by administration of TMX (CREB:*F_(3,20)_* = 7.628, *P < *0.01; *post hoc P < *0.01;* pCREB:F_(3,20)_* = 3.557, *P < *0.05; *post hoc P < *0.05), bringing the values down to those of untreated mice (eg vehicle/non‐fasting in Figure 2A,B). A significant decrease in total CREB levels in PFC was observed from TMX alone in fasting mice (*P < *0.05).

Two‐way ANOVA also indicated that E2 treatment and 9 hours fasting changed the levels of CREB and p‐CREB in the HP (*F*_diet_ *_(1,20)_* = 5.466, *P* < 0.05; *F*_treatment_ *_(1,20)_* = 13.843, *P* < 0.01; *F*_diet × treatment_ *_(1,20)_* = 0.586, *P* = 0.453 in Figure 2C;* F*_diet_ *_(1,20)_* = 39.965, *P* < 0.001; *F*_treatment_ *_(1,20)_* = 9.782, *P* < 0.01; *F*_diet × treatment_ *_(1,20)_* = 0.936, *P* = 0.345 in Figure 2D). *Post hoc* analysis showed that administration of E2 significantly increased the levels of CREB and p‐CREB in non‐fasting mice (*P* < 0.05 in Figure 2C; *P* < 0.01 in Figure 2D). Fasting also increased p‐CREB levels compared with non‐fasting mice (*P* < 0.001 in Figure 2D). One‐way ANOVA showed that effects of E2 with fasting on CREB and p‐CREB levels were reversed by TMX (CREB:*F_(3,20)_* = 5.321, *P < *0.05; *post hoc P* < 0.05 in Figure 2G; p‐CREB: *F_(3,20)_* = 8.532, *P < *0.001; *post hoc P* < 0.001 in Figure 2H), again returning the values to control levels.

### 3.3 Effects of fasting, E2 and TMX on BDNF protein levels in the brain

As shown in Figure 2I,J, both E2 treatment and 9 hours fasting increased the levels of BDNF in PFC and HP (*F*_diet_ *_(1,20)_* = 15.888, *P* < 0.01; *F*_treatment_ *_(1,20)_* = 10.207, *P* < 0.01; *F*_diet × treatment_ *_(1,20)_* = 1.086, *P* = 0.31 in Figure 2I; *F*_diet_ *_(1,20)_* = 38.636, *P* < 0.001; *F*_treatment_ *_(1,20)_* = 20.909, *P* < 0.001; *F*_diet × treatment_ *_(1,20)_* = 0.084, *P* = 0.775 in Figure 2J). *Post hoc* comparisons between groups showed that the 9 hours fasting significantly increased levels of BDNF compared with the non‐fasting/VEH group both in the PFC (*P* < 0.01) and the HP (*P < *0.001). E2 treatment also significantly increased levels of BDNF compared with non‐fasting/VEH group both in the PFC and the HP (*P* < 0.01 in the PFC, and *P* < 0.01 in the HP). Effects of E2 with fasting on BDNF levels in both the PFC and HP were reversed by TMX, as shown in Figure 2K (one way ANOVA, *F_(3,20)_* = 3.257, *P* < 0.05) and Figure 2L (one way ANOVA, *F_(3,20)_* = 9.087, *P* < 0.001). *Post hoc* comparisons between groups showed that TMX administration inhibited the combined effects of E2 and fasting in the PFC (*P < *0.05) and HP (*P < *0.001), returning levels to baseline. However, although TMX also slightly reduced BDNF levels that were elevated by fasting alone, no significant differences were observed.

### 3.4 Effects of acute fasting, E2 and TMX on cell proliferation in the SGZ of the hippocampal dentate gyrus

**Figure 3:**
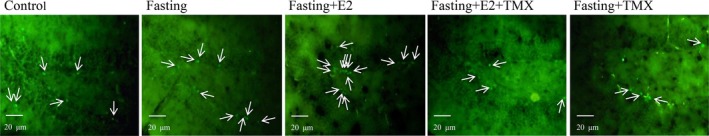
Fasting increased the number of BrdU‐ positive cells in the hippocampal dentate gyrus, E2 treatment had additional effects when combined with fasting, and the effect was antagonized by TMX. N = 6 per group. Representative fluorescence photomicrographs of these effects on the number of BrdU‐positive cells of the dentate gyrus. E2: 17 β‐estradiol; TMX: tamoxifen

As shown in Figure 3, 9 hours fasting increased the number of BrdU‐positive cells in the SGZ of the hippocampal dentate gyrus. In addition, E2 treatment produced additive effects with fasting in the increase in BrdU expression. This effect was antagonized by TMX.

### 3.5 Effects of acute fasting, E2 and TMX on serum ghrelin, E2 and CORT levels

**Figure 4:**
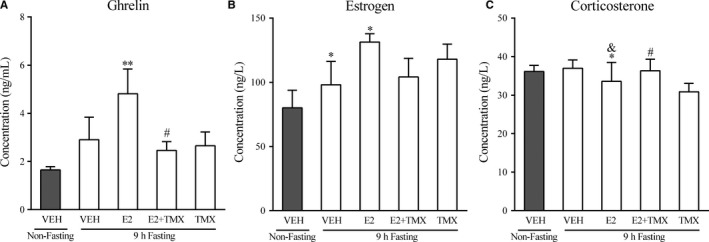
Effects of acute 9 h fasting, E2 and TMX, on serum ghrelin, E2 and CORT levels. Columns represent mean ± SEM. * *P* < 0.05; ** *P* < 0.01 compared with the non‐fasting control (vehicle) group. & *P* < 0.05 compared with the fasting group. # *P* < 0.05 compared with the E2 + fasting group. VEH: vehicle; E2: 17 β‐estradiol; TMX: tamoxifen

A one‐way ANOVA revealed a significant difference in ghrelin (Figure 4A, *F_(4,20)_ =* 3.572, *P < *0.05), E2(Figure 4B, *F_(4,15)_ =* 3.783, *P* < 0.05) and CORT (Figure 4C, *F_(4,25)_* *=* 4.364, *P < *0.01) levels between groups. Although fasting alone did not produce a significant increase in ghrelin, *post hoc* comparisons revealed that fasting combined with estrogen did increase the level of ghrelin compared with the non‐fasting group (*P < *0.01). These effects of E2 in fasting mice were partially reversed by TMX administration (*P < *0.05). Figure 4B shows that after 9 hours fasting, the level of E2 was enhanced significantly (*P < *0.05), and E2 produced additive effects with fasting, increasing E2 levels. However, there were no substantial difference after TMX treatment. Figure 4C shows that after the E2 treatment in fasting mice, the CORT level was significantly reduced compared with non‐fasting/VEH group (*P < *0.05) and fasting/VEH group (*P < *0.05) and these effects were inhibited by TMX administration (*P < *0.05).

### 3.6 Identification of differential gene expression in the PFC and HP in mice after fasting or E2 treatment

**Figure 5:**
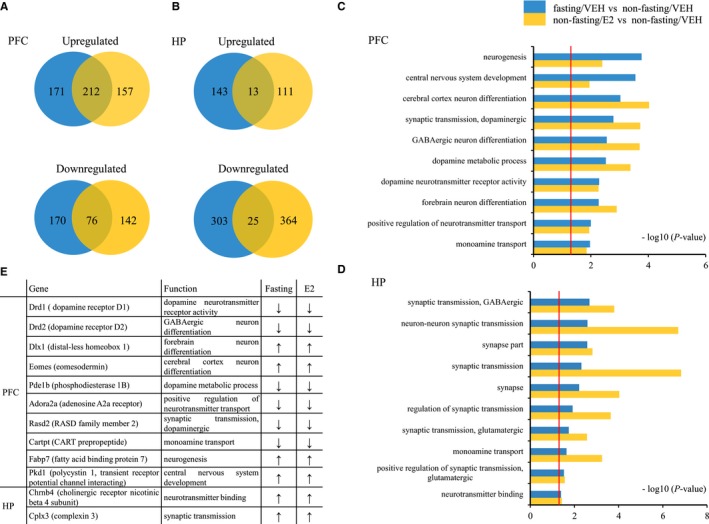
DEGs and Gene Ontology (GO) analyses of fasting and E2 treatment in the PFC and HP in mice. Both treatments were compared with the non‐fasting control (vehicle) group. N = 3 per group. Venn diagrams in A and B representing overlap in significantly up‐regulated or down‐regulated mRNAs in the PFC (A) and HP (B) treated with fasting or E2 in mice. Bar graphs at right show the neurological functions that are enriched in DEGs after fasting and E2 treatment in the PFC (C) and HP (D) in mice. The threshold of DEGs and GO enrichment was set to *P* value ≤ 0.05, red line in C and D indicates *P* = 0.05. Table in E shows the representative genes that were up‐ or down‐regulated in the PFC and HP by fasting or E2 treatment. The threshold was set to *P* ≤ 0.05, fold change >1.3 (↑) or fold change <0.83 (↓). PFC: prefrontal cortex; HP: hippocampus; E2: 17 β‐estradiol; VEH: vehicle.

Venn diagrams in Figure 5A,B showed that there was a considerable overlap in genes regulated by fasting and E2 treatment compared to the non‐fasting/VEH group in the PFC and HP. In the PFC, in the fasting/VEH group, 383 transcripts were up‐regulated, and 246 transcripts were down‐regulated when compared to the non‐fasting/VEH group. In the non‐fasting E2 group, 369 transcripts were found to be up‐regulated and 218 transcripts were down‐regulated when compared to the non‐fasting/VEH group. Interestingly, the two treatments had 212 genes that were similarly up‐regulated and 76 genes that were down‐regulated in common. In the HP, in the fasting/VEH group, 156 transcripts were up‐regulated, and 328 transcripts were down‐regulated when compared the non‐fasting/VEH group. In the non‐fasting E2 group, 124 transcripts were found to be up‐regulated and 389 transcripts were down‐regulated when compared to the non‐fasting/VEH group. Interestingly, the proportion of co‐regulated genes was smaller, with the two different treatments having 13 genes that were similarly up‐regulated and 25 genes that were down‐regulated.

### 3.7 Gene Ontology (GO) enrichment in the PFC and HP in mice after fasting or E2 treatment

Fasting produced effects that were enriched for genes involved in neurogenesis (Figure 5C: *P < *0.001 in fasting/VEH vs non‐fasting/VEH groups), central nervous system development and cerebral cortex neuron differentiation. E2 treatment in VEH mice produced effects that were enriched for genes involved in central cortex neuron differentiation, dopaminergic synaptic transmission and GABAergic neuron differentiation (Figure 5C: *P < *0.001 in non‐fasting/E2 vs non‐fasting/VEH groups). Significant enrichment was produced by fasting and E2 for all these functions in PFC, even though some were more affected by one treatment or the other. Figure 5D shows the gene ontology analysis in the HP for fasting and E2 treatments. Again, there were substantial overlaps between these effects even though one or the other treatment had greater effects on particular gene sets. Fasting had more effect on GABAergic synaptic transmission and neuron‐neuron synaptic transmission (*P < *0.01 in fasting/VEH vs non‐fasting/VEH groups). Neuron‐neuron synaptic transmission was also the gene class most affected by E2 in VEH mice (*P < *0.0001 in non‐fasting/E2 vs non‐fasting/VEH groups), along with synaptic transmission and synaptic genes. Overall, different aspects of synaptic transmission were the most common biological processes affected by fasting and E2 in both the PFC and HP, particularly function related to GABAergic, glutamatergic and dopaminergic functions.

Table in Figure 5E shows examples of genes that are regulated in common in fasting/VEH and non‐fasting/E2 mice in the PFC and HP from these gene categories identified in GO analyses. Down‐regulated genes included *Drd1*, *Drd2*, *Pde1b*, Cartpt, *Adora2a and Rasd2* in the PFC. Up‐regulated genes in the PFC included *Dlx1*, *Eomes*, *Fabp7* and *Pkd1*, while *Cplx3* and *Chrnb4* were up‐regulated in HP.

## 4 DISCUSSION

Our previous studies demonstrated that acute 9 hours fasting has antidepressant‐like effects in male mice.^7^ In this study, we find that fasting reduced immobility time in female mice, and that E2 produces an additive effect on immobility time without changing locomotor activity. These antidepressant‐like effects were blocked by TMX, which is a mixed estrogen receptor antagonist.^18^ This suggests that the estrogen receptor is involved in antidepressant‐like effects of fasting as well as E2.

To further investigate the mechanisms of the antidepressant‐like effects of fasting and E2, Western blotting examined the CREB‐BDNF pathway, which has repeatedly been associated with the effects of pharmacological treatments that produce antidepressant‐like behavioural changes. Nine hours fasting, as well as E2 treatment, enhanced total CREB and p‐CREB levels in both PFC and HP. These effects were blocked by TMX, suggesting that both effects involved estrogen receptors. These findings support previous findings that E2 increases CREB protein expression, as well as p‐CREB expression in the HP.^19^ Similarly, these results are consistent with another study that reported p‐CREB was increased in chronic caloric restricted mice.^20^ Downstream from these changes in p‐CREB and CREB expression, BDNF protein levels were also increased after 9 hours fasting in the PFC and HP, and E2 produced comparable effects. TMX treatment reversed these effects, again implicating estrogen receptors in both the effects of fasting and E2. Estrogen has been suggested to be one of factors influencing levels of BDNF mRNA and protein expression 21 and calorie restriction up‐regulates BDNF/TrkB expression in the HP of dietary‐induced obese rats.^22^ Based on the current CREB‐BDNF signalling results, we have been suggested that the CREB‐BDNF pathway is at least partly involved in the antidepressant‐like effects of acute fasting and E2 found here.

Moreover, increased BDNF expression by antidepressants is thought to influence another fundamental mechanism of these effects: neurogenesis,^23^ which also appears to play a role in the antidepressant‐like effects of fasting and E2. BrdU‐positive cells were increased by 9 hours fasting, and the addition of E2 further increased the number of BrdU‐positive cells compared with the non‐fasting group or the fasting alone group. Moreover, this effect was also blocked by TMX, implicating a role of E2 in these changes as well. It has been reported that estrogen deficiency reduces adult hippocampal neurogenesis,^24^ and depression is of course linked to a variety of conditions that involve reduced or altered estrogen function, including post‐partum depression 25 and menopause.^26^ Dietary restriction is also known to alter the process of neurogenesis.^27^ Taken together, these data suggest that neurogenesis may be involved in estrogen‐mediated antidepressant‐like effects of acute fasting in female mice.

To further elucidate the mechanisms of the antidepressant‐like effects of fasting and E2, we examined serum ghrelin, estrogen and CORT levels. CORT levels did not appear to be significantly affected by 9 hours fasting treatment. CORT is one of the effector hormones of the hypothalamic‐pituitary‐adrenal (HPA) axis and is mildly increased by a longer period of fasting.^28^ In any case, with respect to the present results, it does not appear that CORT contributes to the antidepressant‐like effects of fasting. By contrast, ghrelin levels were significantly changed by fasting or fasting plus E2. It has been reported that fasting increases ghrelin levels 29 and ghrelin treatment significantly increases estradiol secretion.^30^ Thus, ghrelin secretion may account for the apparent estrogen mediation of the effects of fasting. This hypothesis is supported by the ability of TMX to antagonize the effects of fasting. Moreover, increases in estradiol secretion do not occur in GHSR 1a knockout (Ghsr1a‐/‐) mice after calorie restriction,^6^ which is a further indication of this ghrelin‐estrogen connection. These results suggest that ghrelin may be related to the antidepressant‐like effects of calorie restriction, but the direction of these effects is open to some question. After the addition of E2, ghrelin levels were higher than in the non‐fasting control group and TMX blocked the enhancement of ghrelin level compared with fasting plus E2. Indeed, effects of estrogen on ghrelin levels have been reported by previous studies.^31^ Estrogen treatment significantly increased the level of ghrelin production and ghrelin mRNA expression, and ICI‐182 780 (a pure ER antagonist) completely reversed the effect of estrogen.^31^ E2 levels were also changed by fasting or fasting plus estrogen. These effects were also blocked by TMX. Thus, it appears that ghrelin and E2 play roles in the antidepressant‐like effects of fasting and E2, but the exact nature of this interaction remains to be fully elucidated and may be bidirectional.

Besides cellular and molecular mechanisms of fasting and estrogen, genomic mechanisms were also examined here. RNA‐seq has been used to explore differences in gene expression in depression.^32^ Transcriptome analysis was performed in calorie‐restricted rats to explore the mechanisms of calorie restriction on neuroprotection and its positive effects on ageing‐related declines in neural function.^33^ Here, fasting and E2 treatments were found to have substantial overlaps in their effects on gene expression, particularly in the PFC, where 212 genes were up‐regulated and 76 genes were down‐regulated in common. In the HP, fasting and E2 treatments had less overlap, with 13 genes dually up‐regulated and 25 genes dually down‐regulated. The PFC thus contains more co‐expressed DEGs than HP. In addition, the GO analysis found that there were many neurological functions enriched in DEGs after fasting or E2 treatment in both PFC and HP. More affected genes were related to neural and synaptic function, including neurogenesis, central nervous system development, forebrain neuron differentiation and synaptic transmission, especially GABAergic and dopaminergic transmission. It is well known that neuroprotection and changes in synaptic transmission are associated with antidepressant‐like effects.^34^ In PFC, fasting or E2 co‐treatment down‐regulated phosphodiesterase‐1b (*Pde1b*). PDE1 is a dual substrate for cAMP and cGMP and is found in areas rich in dopamine (DA). It has been reported that *Pde1b* knockout mice are resistant to forced swim and tail suspension induced immobility.^35^ Fasting or E2 treatment also down‐regulated the adenosine A2a receptor (*Adora2a*). ADORA2A in the amygdala has a role in anxiety.^36^ RASD Family Member 2 (*Rasd2*), also down‐regulated in the PFC of fasting or E2 treated mice, is also affected by chronic stress in the PFC of mice.^37^ Complexin 3 (*Cplx3*) was induced in HP by both fasting and estrogen. It has been reported that antidepressants increase *Cplx3* in rat HP.^38^ Taken together, we found that fasting and E2 have substantially co‐regulated gene expression in the PFC and HP, affecting similar neural functions, and in a manner consistent with other antidepressant treatments. Co‐regulated gene expression and their effects on neural function in these brain regions may be related to another mechanism of the additive antidepressant‐like effects of fasting and E2.

Estradiol replacement therapy is an effective treatment of depression for perimenopausal women,^39^ as well as some other conditions that involve impaired estrogen function. The current results would suggest that fasting may be an adjunctive treatment to hormone replacement therapy for postmenopausal women with depressive symptoms, or other conditions in which estrogen function is disrupted, resulting in depressive symptomatology.

## 5 CONCLUSION

In conclusion, our data show that acute fasting produces antidepressant‐like effects in female mice and E2 produces additive antidepressant‐like effects when combined with fasting. The additional effectiveness of E2 may be related to neuroplasticity by increasing of the CREB‐BDNF and neurogenesis. Moreover, serum ghrelin and estrogen are also increased by fasting and E2 and may be important mediators of these effects. Moreover, the results of RNA‐seq analysis suggest that fasting and E2 co‐regulate similar gene targets that affect neural and synaptic functions in the PFC and HP. In summary, we conclude that E2 produces additive antidepressant‐like effects with fasting, and that fasting might be used as an adjunct to estrogen replacement therapy.

## DISCLOSURE STATEMENT

The authors declare no conflict of interest.

## AUTHORS’ CONTRIBUTION

RJC, BZR and BJL conceived and designed the experiments. PW, JF, WY and KZ contributed to the acquisition of data. RJC and BJL analysed and interpreted the data. BJL and RJC contributed to drafting the article. All authors have revised the manuscript critically for important intellectual content and approved the final version to be published.
